# Amino acid-induced regulation of hepatocyte growth: possible role of Drosha

**DOI:** 10.1038/s41419-019-1779-7

**Published:** 2019-07-22

**Authors:** Gaia Fabris, Olivier Dumortier, Didier F. Pisani, Nadine Gautier, Emmanuel Van Obberghen

**Affiliations:** 1 Université Côte d’Azur, Inserm, CNRS, IRCAN, Nice, France; 2 Université Côte d’Azur, CNRS, LP2M Nice, France; 3 Université Côte d’Azur, CNRS, Inserm, iBV, Nice, France; 4 Université Côte d’Azur, CHU, Inserm, CNRS, IRCAN, Nice, France; 5 Université Côte d’Azur, CHU, CNRS, LP2M Nice, France

**Keywords:** Cell signalling, Metabolism

## Abstract

In an adult healthy liver, hepatocytes are in a quiescent stage unless a physical injury, such as ablation, or a toxic attack occur. Indeed, to maintain their crucial organismal homeostatic role, the damaged or remaining hepatocytes will start proliferating to restore their functional mass. One of the limiting conditions for cell proliferation is amino-acid availability, necessary both for the synthesis of proteins important for cell growth and division, and for the activation of the mTOR pathway, known for its considerable role in the regulation of cell proliferation. The overarching aim of our present work was to investigate the role of amino acids in the regulation of the switch between quiescence and growth of adult hepatocytes. To do so we used non-confluent primary adult rat hepatocytes as a model of partially ablated liver. We discovered that the absence of amino acids induces in primary rat hepatocytes the entrance in a quiescence state together with an increase in Drosha protein, which does not involve the mTOR pathway. Conversely, Drosha knockdown allows the hepatocytes, quiescent after amino-acid deprivation, to proliferate again. Further, hepatocyte proliferation appears to be independent of miRNAs, the canonical downstream partners of Drosha. Taken together, our observations reveal an intriguing non-canonical action of Drosha in the control of growth regulation of adult hepatocytes responding to a nutritional strain, and they may help to design novel preventive and/or therapeutic approaches for hepatic failure.

## Introduction

The liver is one of the eminently important organs in the regulation of organismal homeostasis in vertebrates, as it exerts and orchestrates multiple essential biological functions, including all fuel metabolism, detoxification of intrinsic and extrinsic substances and production of numerous proteins with diverse extra-hepatic actions. Proteins, representing ~15% of body mass in healthy adults^1^, have major roles not only as constituents of all cells, but also as molecules enabling cells to function, grow, and differentiate. Therefore, the availability of amino acids (aa), the building blocks of proteins, is crucial for the existence of life itself. The liver has a pivotal role in aa metabolism, being a chief actor in systemic protein synthesis and degradation. Indeed, 60% of the aa taken up by daily diet are destined to be catabolized and take part to the hepatic urea cycle^2,3^. Further, the liver uses aa for gluconeogenesis during starvation^4^ and contributes to plasma aa homeostasis^5^. One of the remarkable features of adult liver is its great regenerative capacities after partial ablation or injuries^6,7^. The liver is composed of parenchymal cells, i.e., hepatocytes, and non-parenchymal cells, including sinusoidal endothelial cells, cholangiocytes, Kupffer cells, and stellate cells. Hepatocytes constitute the most abundant cell type in the liver, accounting for 80% of liver mass, and they perform the majority of the functions mentioned^8^. In an adult healthy liver, hepatocytes show an extremely low proliferation rate as they remain in quiescence until an aggression occurs, which induces entrance in the cell cycle^9,10^. One of the conditions necessary for cell proliferation is an increment in protein synthesis, which allows cells to grow and divide. Aa availability, aside of being obviously indispensable for protein synthesis, has also an essential role in regulating cell proliferation through the activation of mTOR and its downstream S6 kinase^11,12^.

Even though hepatocyte proliferation during liver regeneration is well characterized, yawning gaps exist concerning our understanding of its regulation by aa. The overarching aim of our work was to study how aa participate in the control of the switch between quiescence and growth of adult hepatocytes. By using non-confluent primary adult rat hepatocytes, we mimicked the condition of active proliferation after an injury, thus being able to investigate the regulation of hepatocyte growth capacity by aa. We show that the aa lack induces a quiescence state coupled to increased expression of Drosha, a major actor in microRNA (miRNA) biogenesis. Further, siRNA-induced reduction of Drosha restores proliferation in aa absence, whereas the decrement in Ago2, necessary for miRNA functioning, does not. Collectively, our findings unveil a newly identified non-canonical role of Drosha in regulating the response of adult hepatocytes to aa availability and could contribute to innovative approaches to prevent and/or treat hepatic failure.

## Results

### Amino-acid deprivation blocks primary rat hepatocyte proliferation and induces a metabolic rest without affecting the mitochondria

**Fig. 1:**
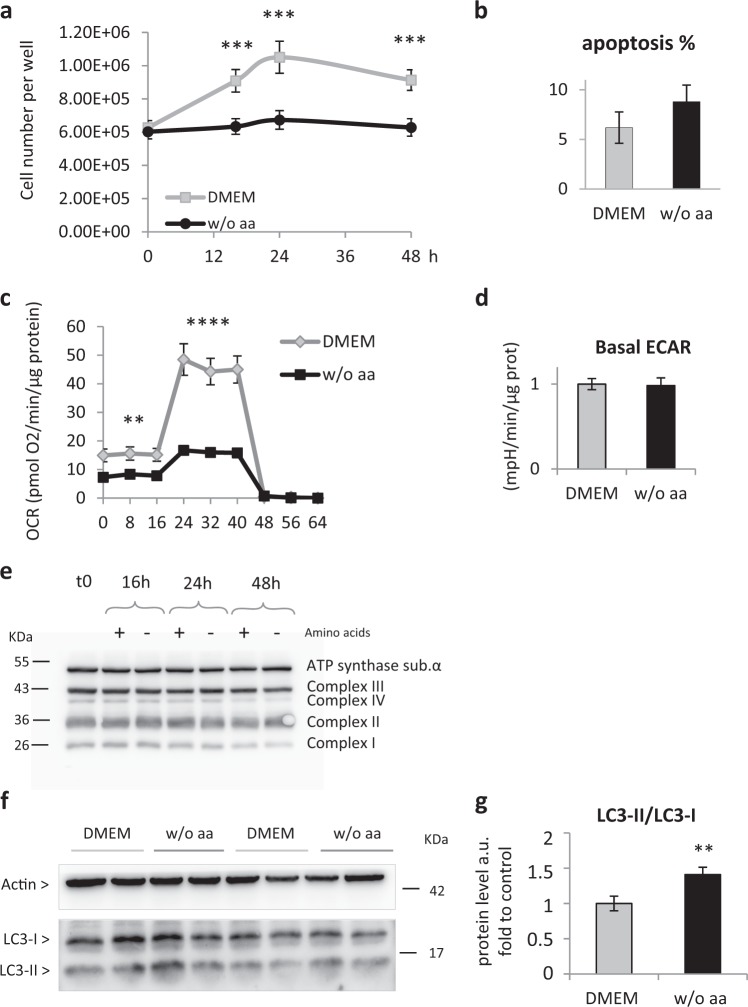
AaD in primary adult rat hepatocytes blocks cell proliferation and reduces mitochondrial metabolism without affecting either the oxphos complexes or the number of mitochondria. **a** Cell counting of primary adult rat hepatocytes in presence of aa (DMEM) and in absence of aa (w/o aa) at different time points. Values are means ± SEM (*n* = 3). ***p < 0.0005. **b** Quantification of primary adult rat hepatocyte’s apoptotic rate in DMEM and after 48 h aaD (w/o aa) by Annexin V FACS analysis. Results are expressed in percentage of Annexin V-positive cells over the total number of events registered (i.e., cells counted) by the flow cytometer. Values are means ± SEM (*n* = 3). **c** Mitochondrial oxygen consumption rate (OCR) from primary adult rat hepatocytes after 48 h from plating in DMEM or w/o aa. Maximal OCR was determined using FCCP (2 µm). OCR is reported in the unit of picomoles per minute. Values are means ± SEM (*n* = 16 replicates from two independent rats). **p < 0.005, ****p < 0.0001. **d** Basal extracellular acidification rate (ECAR) from primary adult rat hepatocytes after 48 h from plating in DMEM or w/o aa. ECAR is reported in milli-pH units (mpH) per minute. Values are means ± SEM (*n* = 16 replicates from two independent rats). **e** Western blot with antibody to the oxphos complexes of cell lysates obtained from primary rat hepatocytes after isolation (t0) and cultured for 16 h, 24 h, and 48 h either in DMEM or in medium w/o aa. (*n* = 3). **f** Western blot analysis of LC3-I/II in primary adult rat hepatocytes after keeping them in culture for 48 h in DMEM and in medium w/o aa; cells were treated with 25 µm chloroquine for 1 h before protein extraction. For quantification, **g** each band was normalized by the actin band and the normalized values of LC3-II were then divided by LC3-I values. Values are means ± SEM (*n* = 3). **p < 0.01

To investigate the effect of total aa deprivation (aaD) on primary adult rat hepatocytes, we cultured them in aa presence or absence. The number of cells grown in a full-aa medium Dulbecco’s Modified Eagle Medium (DMEM) reaches a maximum within 24 h following cell isolation and remained constant thereafter up to 48 h. In contrast, the number of hepatocytes cultured without aa was steady up to 48 h, suggesting that they do not grow (Fig. 1a). Further, they maintained their original phenotype as no differences in the expression of key hepatocyte markers were observed after 48 h aaD (not shown). In addition, the aa-deprived hepatocyte population appears to be more stable after 48 h, as their number decreases slower compared to hepatocytes in complete DMEM. Indeed, after 72 h aaD the cell number decreases only by 30% against 70% in cells cultured in full-aa medium (Fig. S1a). To investigate whether this difference was owing to changes in cell proliferation or in cell death, we analyzed apoptosis after 48 h culture. We found that aaD does not increase apoptosis (Fig. 1b, S1b, c), suggesting that the stable cell number in aaD is likely to be owing to proliferation arrest. To explore the effect of aaD on cell metabolism, we measured the oxygen consumption rate in aa presence or absence. As illustrated in Fig. 1c, aa lack leads to a twofold decrease in basal mitochondrial respiration and a 2.8-fold reduction in maximal mitochondrial respiration, but no diminished glycolysis in basal condition was found (Fig. 1d). Remarkably, this cannot be explained by differences in the protein level of the OXPHOS complexes (Fig. 1e). Further, both the network and the number of mitochondria per cell appear to be similar in both conditions (Fig. S1d, e). To decrypt in more detail the effect of aaD on primary rat hepatocytes, we investigated the implication of autophagy in aa presence or absence. As expected, a lack of aa leads to an increased LC3-II/LC3-I ratio, indicating augmented autophagy (Fig. 1f, g, S1f). To summarize, aa-deprived primary hepatocytes are growth-arrested and this is associated to blunted mitochondrial metabolism and increased autophagy.

### Amino acid-deprived hepatocytes are able to exit growth arrest after the addition of essential amino acids

**Fig. 2:**
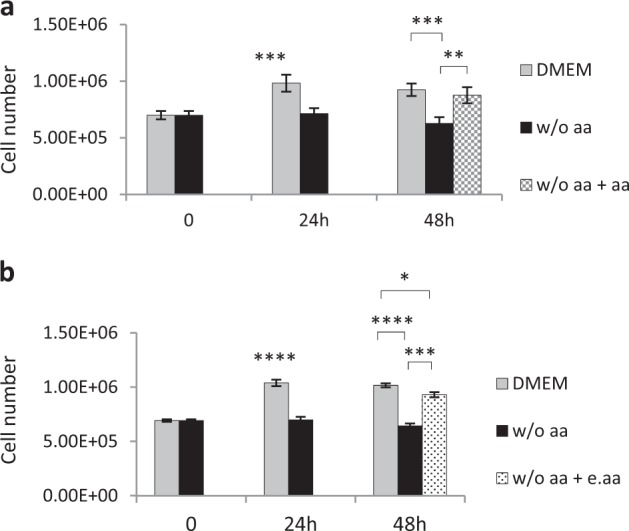
aaD in primary adult rat hepatocytes induces a quiescence state, which can be reversed by aa addition. Cell counting of primary adult rat hepatocytes at t0, 24 h and 48 h after isolation. For the last time point, cells were kept in presence or absence of aa (DMEM; w/o aa) for 48 h, or in medium w/o aa for 24 h with the restoration of total aa **a** or essential aa **b** for the following 24 h. Values are means ± SEM (*n* = 3). *p < 0.05, **p < 0.005, ***p < 0.0005, ****p < 0.0001

Next, we asked whether the hepatocyte growth arrest was reversible or not. To this aim, we cultured primary adult rat hepatocytes for a total of 48 h, the first 24 h in aa absence and the following 24 h with total aa restoration. Cell counting showed that the addition of a full supply of aa (essential and non-essential) to the medium allows hepatocytes to restart to proliferate reaching a level comparable to not depleted hepatocytes (Fig. 2a). Interestingly, the addition of solely essential aa (e.aa) gave partially different results as restoration of the cell number was not completely attained, showing a 10% deficit compared with non-depleted hepatocytes (Fig. 2b). Together, these findings suggest that aaD induces in primary adult rat hepatocytes a quiescence state, which can be reversed by aa addition. However, e.aa apparently do not suffice to generate a full-blown restoration.

### In aa-deprived-growth-arrested hepatocytes the activation of the mTOR pathway is repressed and this inhibition is partially reversed by essential aa

**Fig. 3:**
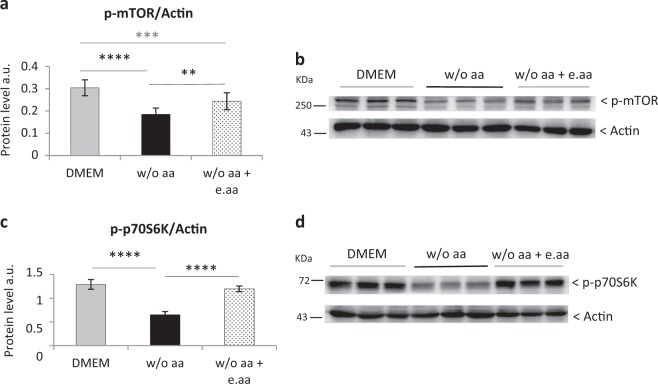
48 h aaD reduces mTOR and p70S6K phosphorylation, and the addition of e.aa to the medium is not sufficient to restore mTOR phosphorylation level. **a**, **b** Western blot analysis **a** and quantification **b** of mTOR phosphorylation after 48 h in DMEM, w/o aa and in medium w/o aa supplemented with essential aa (w/o aa + e.aa). Values are means ± SEM (*n* = 4). **p < 0.01, ***p < 0.0005, ****p < 0.0001. **c**, **d** Western blot analysis **c** and quantification **d** of p70S6K phosphorylation after 48 h in DMEM, w/o aa and w/o aa + e.aa. Values are means ± SEM (*n* = 7). ****p < 0.0001

Considering the importance of aa in the regulation of the nutrient-sensing mechanistic target of rapamycin (*mTOR*) pathway^13,14^ and its role in cell growth^15^, we investigated mTOR activation in primary rat hepatocytes in aa presence or absence. Phosphorylation of both mTOR and its downstream kinase p70S6K are, respectively, reduced by 30% and 50% in aa-deprived cells (Fig. S2e–h). As the total protein level of both kinases is unchanged (Fig. S2a–d), this is compatible with the idea that activation of the mTOR pathway is repressed by aaD. The inhibition of mTOR phosphorylation was not entirely owing to e.aa deprivation, as e.aa addition restores only partially mTOR phosphorylation. In fact, we observed an 18% phosphorylation decrease compared with levels obtained in hepatocytes cultured in DMEM (Fig. 3a, b). Intriguingly, the analysis of p70S6K in the same conditions gave a partially different outcome, as the presence of e.aa in the medium results in p70S6K phosphorylation levels comparable to those seen in the full-aa medium (Fig. 3c, d).

### Essential aa deprivation in primary adult rat hepatocytes leads to an increase in Drosha protein level

**Fig. 4:**
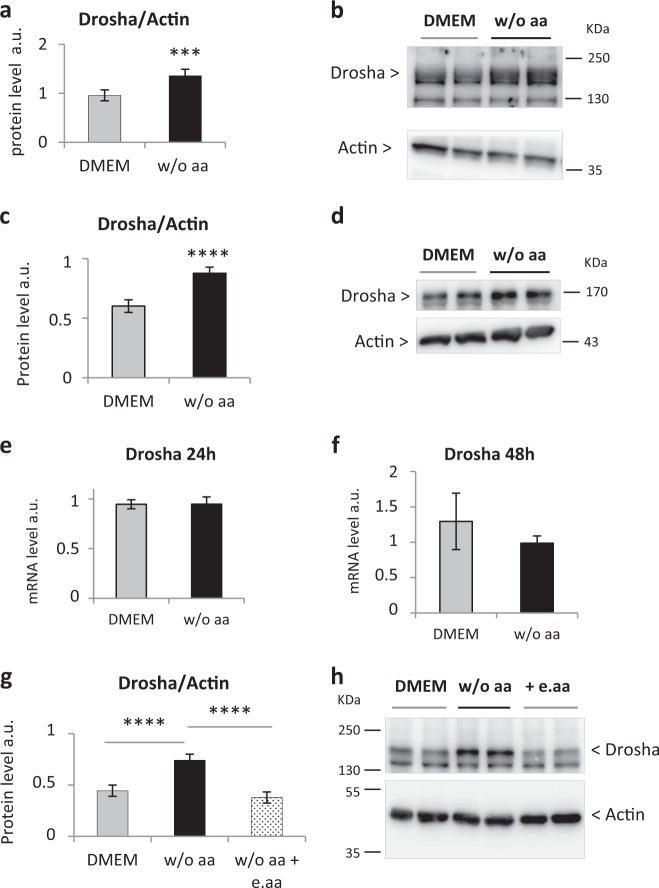
Essential aaD significantly increases Drosha protein in primary adult rat hepatocytes. **a**, **b** Western blot quantification **a** and analysis **b** of Drosha protein level after 24 h in a full-aa medium (DMEM) or in medium w/o aa. Values are means ± SEM (*n* = 5). ***p < 0.0005. **c**, **d** Western blot quantification **c** and analysis **d** of Drosha protein level after 48 h in DMEM or medium w/o aa. Values are means ± SEM (*n* = 16). ****p < 0.0001. **e**, **f** Drosha mRNA measurement in primary adult rat hepatocytes: after 24 h **e** or 48 h **f** aaD, RNA was extracted, reverse transcribed and analyzed by quantitative PCR. Gene expression was normalized to the cyclophilin A (CyA) mRNA level. Values are means ± SEM (*n* = 3). **g**, **h** Western blot quantification **g** and analysis **h** of Drosha protein level after 48 h in DMEM, w/o aa and in medium w/o aa supplemented with essential aa (w/o aa + e.aa). Values are means ± SEM (*n* = 7). ****p < 0.0001

Using murine embryonic fibroblasts deprived of glucose and amino acids, Ye et al.^16^ revealed the involvement of mTOR in miRNA biogenesis through an increase in Drosha protein. To delve into the mechanisms implicated in the effects of aaD on hepatocyte metabolism, we investigated the effect of aa restriction on Drosha. Similarly to Ye et al., both 24 and 48 h aaD in primary rat hepatocytes lead to a 30% increase in Drosha protein (Fig. 4a–d) without affecting its mRNA level (Fig. 4e, f). Interestingly, e.aa are sufficient to counteract the increment in Drosha protein and keep it at the same level observed in full-aa medium (Fig. 4g, h). To summarize, in addition to enabling hepatocytes to exit from quiescence, e.aa are able to prevent the increase in the Drosha protein.

### Neither short- nor long-term rapamycin treatment alters the Drosha protein level in primary adult rat hepatocytes

**Fig. 5:**
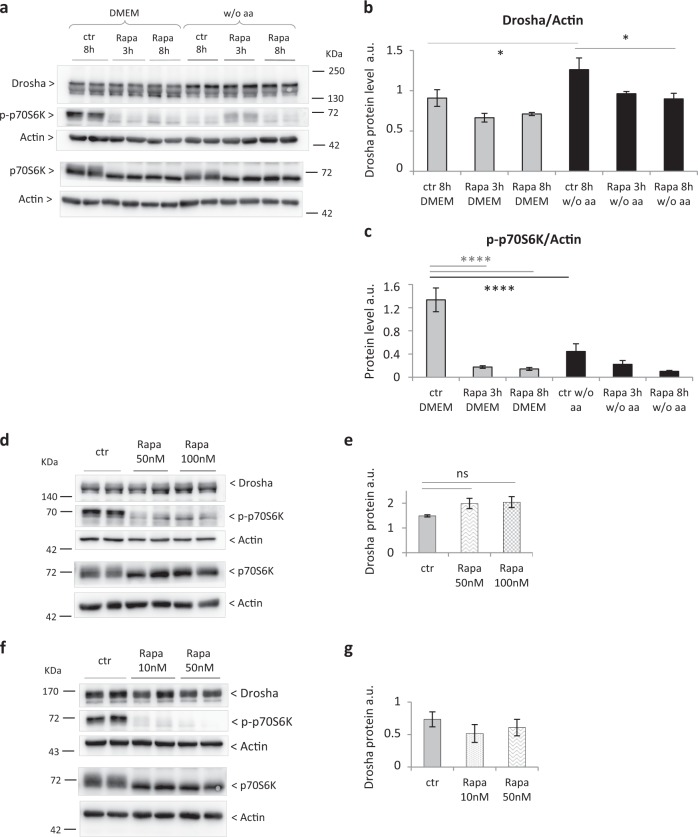
Neither short- nor long-term rapamycin treatments are able to reproduce the increase of Drosha observed in aa-deprived primary adult rat hepatocytes. **a** Western blot analysis of Drosha protein level, p70S6K total protein, and p70S6K phosphorylation. **b** Western blot quantification of Drosha protein in primary adult rat hepatocytes after 48 h aaD without any treatment (Rapa ctr), after 3 h and 8 h rapamycin treatment (100 nm) (Rapa 3 h, Rapa 8 h). Rapamycin was solubilized in DMSO, which was present in the rapamycin and control condition at a final concentration of 0.05 µl/mL. Values are means ± SEM (*n* = 3). *p < 0.05. **c** Western blot quantification of p70S6k phosphorylation in primary adult rat hepatocytes after 48 h aaD without any treatment (Rapa ctr), after 3 h and 8 h rapamycin treatment (100 nm) (Rapa 3 h, Rapa 8 h). Note that p70S6K phosphorylation was measured as read out of rapamycin effect on mTOR activation. Values are means ± SEM (*n* = 3). ****p < 0.0001. **d** Western blot analysis of Drosha protein level, p70S6K total protein and p70S6K phosphorylation and **e** western blot quantification of Drosha protein in aa-deprived hepatocytes without any treatment (ctr) and rapamycin treatment for 24 h. Values are means ± SEM (*n* = 3). **f** Western blot analysis of Drosha protein level, p70S6K total protein, and p70S6K phosphorylation and **f** western blot quantification of Drosha protein in aa-deprived hepatocytes without any treatment (ctr) and rapamycin treatment for 48 h. Values are means ± SEM (*n* = 5)

To assess the involvement of the mTOR pathway in Drosha modulation we treated primary adult rat hepatocytes with rapamycin. However, the specific inhibition of mTORC1 through a short-term treatment did not reveal an increment of Drosha (Fig. 5a, b), although it was sufficient for the inhibition of p70S6K phosphorylation (Fig. 5a–c, S3a). In contrast, an 8 h exposure resulted in a significant decrease in Drosha (Fig. 5a, b). In addition, 24 and 48 h treatment, likely responsible for the inhibition of both mTORC1 and mTORC2, did not lead to changes in Drosha protein (Fig. 5d–g, S3b, c). Together, our findings would indicate that in primary rat hepatocytes the mTOR pathway is not involved in the Drosha increase induced by aa depletion.

### AaD in primary adult rat hepatocytes increases Drosha protein stability

**Fig. 6:**
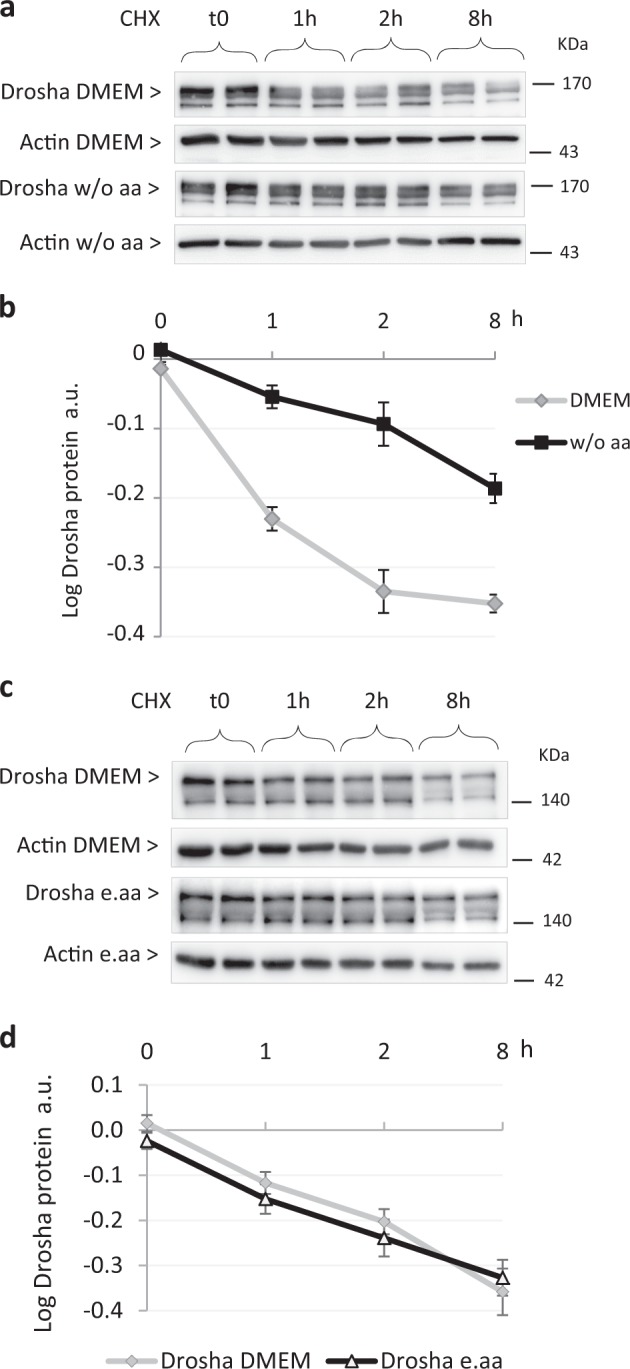
48 h aaD in primary adult rat hepatocytes increases Drosha protein stability. **a** Western blot analysis and **b** quantification of Drosha protein level in primary adult rat hepatocytes cultured in full-aa medium (DMEM) or in absence of aa (w/o aa); **c** Western blot analysis and **d** quantification of Drosha protein level in primary adult rat hepatocytes cultured in full-aa medium (DMEM) or in medium without aa supplemented with essential aa (w/o aa+e.aa). The protein content of cells isolated from the same liver was analyzed just before treatment (t0) or after 1 h, 2 h, and 8 h from the addition of cycloheximide (CHX) (50 µm). Values are means ± SEM (*n* = 3). *p < 0.05, ***p < 0.0005

To further untangle the mechanism underlying the Drosha increase, we determined its half-life in aa presence or absence. As illustrated in Fig. 6a, b, the lack of aa (essential and non-essential) in primary rat hepatocytes results in a slower degradation rate of Drosha compared with cells grown in a full-aa medium, the corresponding half-life of Drosha in medium w/o aa and in full-aa medium being, respectively, 1.9 h and 0.6 h. Interestingly, the comparison of Drosha half-life between cells grown in full-aa medium and those supplemented with e.aa shows no difference (Fig. 6c, d). These results support the notion that the augmentation of Drosha dependent on total aaD is owing, at least partly, to an increase in its protein stability compared with the control condition.

### Drosha knockdown allows cell proliferation in aa-deprived hepatocytes without affecting mitochondrial metabolism and autophagy

**Fig. 7:**
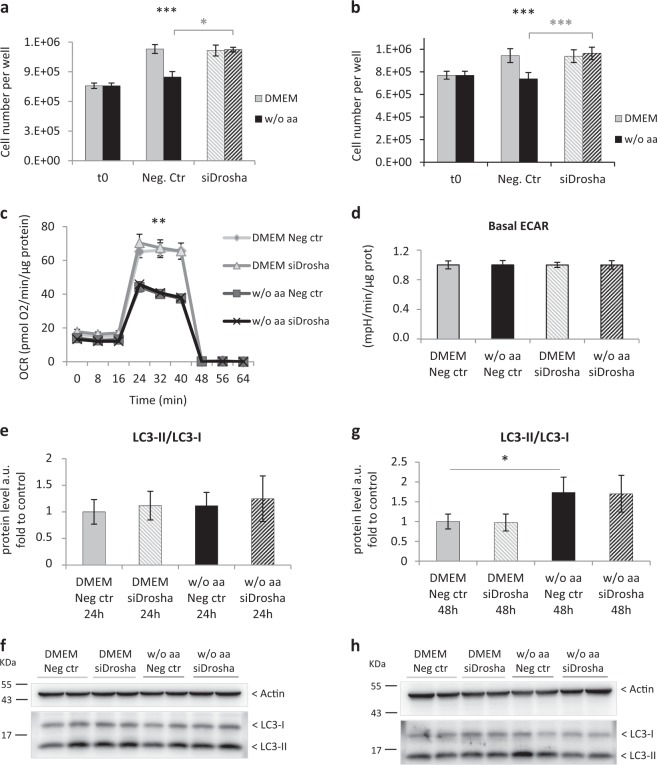
Drosha knockdown restores cell proliferation in aa-deprived primary adult hepatocytes without affecting oxygen consumption and autophagy. **a**, **b** Cell counting of primary rat hepatocytes transfected with 50 nmol/L negative control-siRNA (Neg. Ctr) or Drosha-siRNA (siDrosha) for 24 h **a** and 48 h **b**, in full-aa medium (DMEM) or in absence of aa (w/o aa). Values are means ± SEM (*n* = 3). *p < 0.05, ***p < 0.0005. **c**, **d** Mitochondrial oxygen consumption rate (OCR) **c** and basal extracellular acidification rate (ECAR) **d** from primary rat hepatocytes cultured in DMEM or w/o aa and transfected with 50 nmol/L negative control-siRNA (Neg. Ctr) or Drosha-siRNA (siDrosha) for 48 h. Maximal OCR was determined using FCCP (2 µm). OCR is reported in the unit of picomoles per minute and ECAR is reported in milli-pH units (mpH) per minute. Values are means ± SEM (*n* = 11 replicates from two independent rats). **p < 0.005. **e**–**h** Western blot quantification and analysis of LC3-I/II in primary adult rat hepatocytes transfected with 50 nmol/L negative control-siRNA (Neg. Ctr) or Drosha-siRNA (siDrosha) and kept in culture for 24 h **e**, **f** or 48 h **g**, **h** either in DMEM or in medium w/o aa; cells were treated with 25 µm chloroquine for 1 h or 4 h before protein extraction. Values are means ± SEM (*n* = 3). *p < 0.05

To clarify Drosha’s role in aa-deprived hepatocytes we knocked down its expression using siRNA. As illustrated in Fig. 7a, Drosha knockdown in primary adult rat hepatocytes allows proliferation already after 24 h aaD. Likewise, hepatocytes exposed to 48 h aaD and having an induced reduction of Drosha, display normal growth (Fig. 7b). Considering the proliferating capability of hepatocytes with Drosha knocked down in aa absence, we wondered whether Drosha could also affect cell metabolism. However, the analysis of OCR in presence or absence of Drosha after 48 h aaD did not reveal any change either in basal and maximal mitochondrial respiration (Fig. 7c), or in glycolysis in basal conditions (Fig. 7d). Therefore, Drosha appears to be involved in the regulation of primary adult rat hepatocyte proliferation but exerts no detectable effect on their oxygen consumption in presence or absence of aa.

We revealed earlier that, in absence of Drosha, aa-deprived hepatocytes are able to grow. It is accepted that autophagy supports cell survival under nutrient and energy deprivation and its activation is classically regulated through the inhibition of the mTOR pathway. In light of this we investigated an alternative regulation mechanism for this process through increased Drosha. The analysis of LC3 cleavage demonstrated that Drosha knockdown has no effect on autophagy after 24 h aaD (Fig. 7e, f) and that the increase after 48 h aaD does not depend on Drosha (Fig. 7g, h). Together, our results do not support Drosha’s involvement in autophagy regulation.

### In primary adult rat hepatocytes deprived of aa the expression of miR-23a/b and miR-27b is increased, but Ago2 knockdown does not affect cell proliferation

**Fig. 8:**
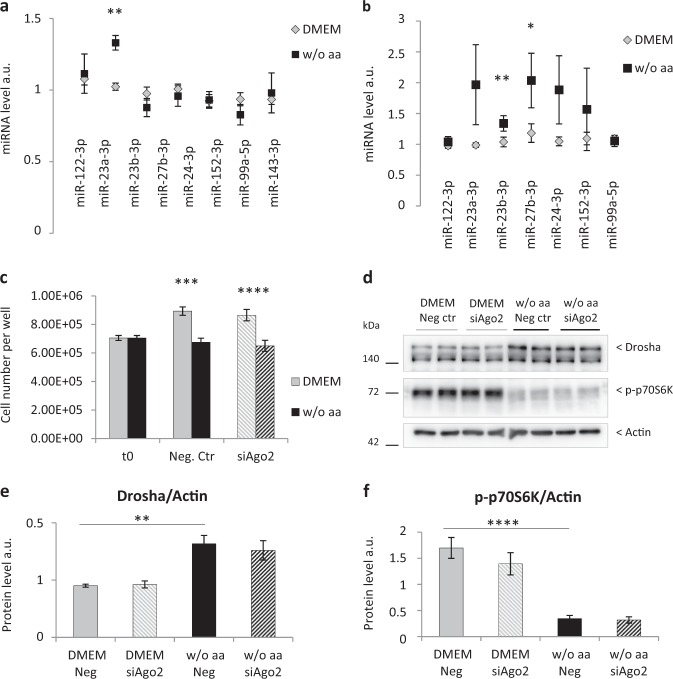
AaD in primary adult rat hepatocytes increases the expression of miR-23a/b and miR-27b, but Ago2 knockdown has no effect either on cell proliferation or on Drosha protein. Analysis of miRNA expression level in primary adult rat hepatocytes after 24 h **a** and 48 h **b** either in full-aa medium (DMEM) or medium w/o aa. RNA was extracted, reverse transcribed and analyzed by Real Time PCR. The expression of miR-23a-3p, -23b-3p, -27b-3p, -24-3p, -152-3p, -99a-5p, -143-3p was normalized to the miR-122-3p transcript level, whereas the expression of miR-122-3p was normalized to miR-99a-5p. Values are means ± SEM (*n* = 3). *p < 0.05, **p < 0.005. **c** Cell counting of primary adult rat hepatocytes transfected with 50 nmol/L negative control-siRNA (Neg. Ctr) or Ago2-siRNA (siAgo2) for 48 h in full-aa medium (DMEM) or in absence of aa (w/o aa). Values are means ± SEM (*n* = 3). ***p < 0.0001, ****p < 0.00001. **d** Western blot analysis of Drosha protein level and p70S6K phosphorylation in primary adult rat hepatocytes transfected with 50 nmol/L negative control-siRNA (Neg. Ctr) or Ago2-siRNA (siAgo2) for 48 h in full-aa medium (DMEM) or in absence of aa (w/o aa); **e**, **f** western blot quantification of Drosha protein **e** and p70S6K phosphorylation **f** in aa-deprived hepatocytes transfected with Ago2-siRNA or the negative control, compared with hepatocytes in full-aa medium. Values are means ± SEM (*n* = 3). **p < 0.001, ****p < 0.00001

Having observed an increase in Drosha in aa-deprived hepatocytes, we evaluated whether aaD could be linked to a general miRNA upregulation. Therefore, we analyzed the expression of a subset of liver specific and/or enriched miRNAs in aa presence or absence. Among these miRNAs, after 24 h aaD only miR-23a-3p is significantly upregulated, with a 1.3-fold increase compared with control cells (Fig. 8a). Similarly, 48 h aaD significantly augmented only the expression of miR-23b-3p and miR-27b-3p, showing, respectively, a 1.3 and 1.8-fold increment (Fig. 8b).

To approach a possible involvement of miRNAs in the adaptation of hepatocytes to aa restriction, we investigated cell proliferation after blocking miRNA activity. Argonautes are the main components of the RNA-induced silencing complex needed for miRNA functioning. As Ago2 is the only member of the family with catalytic activity^17^, we knocked it down using siRNA. 70% Ago2 reduction in primary adult rat hepatocytes did not impact cell proliferation, either in aa presence or absence (Fig. 8c). In addition, Ago2 knockdown has no effect either on Drosha protein or on p70S6K phosphorylation (Fig. 8d–f, S4a, b). These results suggest that Drosha’s role in the adaptation of hepatocytes to aa restriction is most likely not dependent on miRNA biogenesis.

## Discussion

The effect of nutrient deprivation has been extensively investigated both in vivo and ex vivo as it affects organismal development and all aspects of mature cell physiology and well-being, and as it induces profound changes in the organs involved in fuel homeostasis in adult life. The liver is a key organ in whole-body homeostasis as it impacts on essential functions such as fuel metabolism, production of a myriad of biologically important molecules, and detoxification of harmful intrinsic and foreign substances. To maintain its multiple vital functions the liver is capable to start growing after partial ablation^6^. As expected, this ability strictly depends on the efficiency of the remaining healthy hepatocytes to regenerate and this is influenced by availability of nutrients. Among those, an increase in the demand of aa, as building blocks of proteins, has been described^18^. A recent study showed that protein restriction in mice decreases the volume of both the liver and hepatocytes, and these defects were not completely reversed by aa restoration in the diet^19^. Hence, we can speculate that a deficiency in dietary protein intake could exert a strong and lasting impact on the capacity of recovery in cases of hepatocellular deterioration. Therefore, a deeper understanding of the effect of aa availability on the liver is needed.

### Amino-acid deprivation of primary adult rat hepatocytes leads to quiescence

To delve into the mechanistic underpinnings of adaptation of the liver to aa scarcity we investigated the effect of aa deprivation (aaD) on primary adult rat hepatocytes. We showed that a complete removal of aa from the hepatocyte culture media arrests cell proliferation without increasing the apoptotic rate, but rather leading to the entrance in a quiescence state. This reversible state is likely to protect and preserve the starved hepatocytes, as quiescence often appears when cells are facing a stress which would undermine their homeostatic integrity and survival^20,21^. In a healthy adult liver, normally the hepatocytes show a very low proliferation rate as the great majority of them are blocked in quiescence, or G0^22^. However, when an aggression occurs, healthy hepatocytes exit the resting state and rapidly enter in the cell cycle in a synchronized manner in an attempt to replace the part of the liver damaged or lost^9,10^. In full-aa medium, our primary rat hepatocytes are able to proliferate until they arrive to confluence, between 24 h and 48 h from isolation. In contrast, when cultured in aa absence, they are not capable to start proliferation. Hematopoietic stem cells, known to exist mostly in a quiescent state^23,24^, are characterized by low levels of mitochondrial activity^25,26^. Similarly, we observed that aaD of primary hepatocytes leads to decreased oxygen consumption compatible with quiescence. This drop does not appear to correlate with a switch in cell metabolism, as no change in glycolysis was observed. Moreover, cells manage to exit growth arrest when essential aa are again available and this ability of restarted proliferation is specific of cells in a quiescence state^20,21^.

### The hepatocyte quiescence induced by amino-acid deprivation is owing to increased Drosha protein, which is not linked to activation of the mTOR pathway

One of the main regulators of cell proliferation is the mTOR pathway, which is controlled by multiple stimuli including aa. As expected, we found an inhibition of this pathway when cells were cultured in aa absence. Surprisingly, we observed that the addition of solely e.aa was not entirely sufficient for mTOR phosphorylation restoration, while it completely suffices for p70S6K activation.

In like manner to us, Ye et al. were interested in investigating the effect of nutrient deprivation on the mTOR pathway. Using murine embryonic fibroblasts their reported that glucose and amino-acid deprivation induces an increment in Drosha through the mTOR pathway. This subsequently results in increased miRNA expression and in protection against apoptosis^16^. Interestingly, we found that also in hepatocytes aaD, and specifically e.aa deprivation, results in an augmented Drosha protein level, whereas at variance the mTOR pathway does not appear to control this Drosha increase in our cells.

Our essential finding is that the augmentation in Drosha protein is sufficient to regulate the entrance of adult hepatocytes in a quiescence state when a lack of aa occurs. Indeed, we found that Drosha knockdown counteracts the entrance in quiescence, re-starting cell proliferation. How the inhibition of Drosha allows cells to proliferate in aa absence is extremely intriguing but remains unclear. We hypothesize that, considering the high glucose concentration and the presence of serum in the culture media, proliferation could be supported by alternative energy sources after that Drosha inhibition eliminated hepatocytes growth arrest. We can also imagine that macropinocytosis could occur, providing building blocks for proliferation from the collagen coating the dishes on which the hepatocytes are cultured. In fact it was recently shown that lung fibroblasts are able to increase the internalization of nanoparticles through macropinocytosis when a collagen extracellular matrix is present^27^. It was reported in primary human foreskin fibroblasts that quiescence, induced by serum starvation, is related to enhanced expression of a series of miRNAs, but also to the downregulation of main actors of miRNA biogenesis through augmented autophagy^28^. Among them, Drosha is decreased after aaD of fibroblasts, which differs from what we observed in primary rat hepatocytes. Moreover, although we also show an increase in autophagy following aaD, this did not appear to be linked to the regulation of Drosha. Concerning the mechanism underlying the aaD-induced increment in Drosha protein, it is likely owing to post-transcriptional effects as its mRNA is apparently not augmented, while we found a greater protein stability. A plausible scenario could be an enhanced Drosha acetylation, which prevents Drosha’s ubiquitination and subsequent degradation^29^. Indeed, an increase in the acetylation of enzymes controlling hepatic metabolism is seen in the context of high fat diet^30^, which is associated also with a decrease in aa content in livers^31^.

### The regulatory action of Drosha on hepatocyte proliferation does not appear to involve microRNAs

The most urgent question to be addressed concerning our observations is to understand by which fundamental biological processes Drosha is able to regulate hepatocyte proliferation. Drosha’s canonical role is to participate to miRNA biogenesis through its RNase activity by recognizing specific motifs on double stranded RNA. MiRNA expression is modulated by environmental signals, thus allowing a finely adapted regulation of gene expression and the adjustment of the cells to specific conditions^32–35^. However, in our hepatocytes we did not observe a general effect of aaD on miRNA expression as, among the miRNAs studied, only miR-23a appeared to be augmented after 24 h aaD, and miR-23b and miR-27b upregulated after 48 h. Moreover, the inhibition of miRNA activity through Ago2 knockdown did not affect the hepatocyte proliferation ability, as aa-deprived cells were not capable of growing. While mammals have 4 Argonautes (Ago1–4) participating in miRNA biogenesis^36,37^, the most relevant one with catalytic activity is Ago2^17,38^. Therefore, we do not favor the idea that a compensatory mechanism of the other Argonautes could counteract Ago2 inhibition and maintain miRNA functioning in aaD. Moreover, we found that in growth-arrested hepatocytes Ago2 knockdown resulted in the downregulation of miR-23a/b, miR-27b and miR-24 (Fig. S4c), which were upregulated or unchanged in growth-arrested control hepatocytes (Fig. 8b). These conflicting data do not support an obvious role of those miRNAs in the regulation of primary hepatocyte growth.

To summarize, our key finding is that Drosha participates in the control of the growth of primary adult hepatocytes and that this action does not appear to be mediated by miRNAs, the canonical partners of Drosha. An urgent challenge is to parse out how this non-canonical action of Drosha is initiated by aa deprivation and how it takes place. Be that as it may, altogether, our observations revealing a novel role of Drosha improve our comprehension of the complex intertwined multifactor and multipath network controlling hepatocyte proliferation. Importantly, all advance in the understanding of hepatocyte growth and hence liver regeneration is extremely invaluable as it may contribute to the design of innovative means to protect the liver and/or to regenerate it in disease situations.

## Materials and methods

### Cell culture

All procedures were approved by the Institutional Animal Care and Use Committee at the University Côte d’Azur, Nice, France (CIEPAL-NCE 00500.02). Hepatocytes were isolated from adult male Wistar rats (200–250 g), from Elevage Janvier, Le Genest St. Isle, France) by collagenase dissociation of the liver as described previously^39^. After isolation, the liver was perfused with 0.15 mg/mL Collagenase Clostridium histolyticum Type IV (Sigma) through the portal vein. Cells were mechanically isolated, centrifuged at 670 g for 1 min and resuspended in DMEM (Gibco) supplemented with 1% (w/v) bovine serum albumin (BSA). Hepatocytes were separated from the rest of the cells by gradient centrifugation with Percoll solution (GE Healthcare) diluted with 1/10 of HBSS (Gibco) at 50 × *g* for 5 min. Then the obtained pellet was washed three times with DMEM supplemented by 0.2% (w/v) BSA and 1% (v/v) penicillin–streptomycin mixture (Gibco) and centrifuged at 170 × *g* for 30 sec. The cells were plated in Corning Biocoat six-well or 12-well plates respectively at a final concentration of 10^6^ cells/well and 350,000 cells/well in Waymouth’s medium (Gibco) supplemented with 10% (v/v) fetal bovine serum (Sigma) and 1% (v/v) penicillin–streptomycin mixture, and maintained at 37 °C, 5% CO_2_. After 4 h, the medium was replaced with DMEM w/o aa (Genaxxon bioscience) supplemented with 3.5 g/L d-glucose, 10% (v/v) fetal bovine serum and 1% (v/v) penicillin–streptomycin mixture, with or without the addition of l-glutamine (Lonza), MEM non-essential amino acid (100 × ) solution (Sigma) and MEM amino acids (50 × ) solution (Sigma). Medium was changed every 24 h.

### Cell counting

Primary rat hepatocytes were seeded at 10^6^ cells per well in a six-well plate. 4 h after plating, cells were detached by addition of 500 μL trypsin-ethylenediaminetetraacetic acid (EDTA, 0.05%) (Gibco) per well at different time points (0, 16 h, 24 h, and 48 h). Detached hepatocytes were then collected, centrifuged at 670 g for 1 min and the pellet resuspended in 1 mL fresh medium. In total, 200 μL of cell suspension was finally diluted in 20 mL PBS and cells were manually counted by using a Nageotte chamber.

### Transfection

Primary rat hepatocytes were seeded at 3.5 × 10^4^ cells per well in a twelve-well plate. 4 h after plating, cells were transfected using Lipofectamine RNAiMax (Thermo Fisher Scientific) according to the manufacturer’s guide. Each well was transfected with 50 nmol/L of Drosha-siRNA (ON-TARGETplus Rat Drosha-siRNA—SMARTpool, Dharmacon), Ago2-siRNA (ON-TARGETplus Rat Ago2-siRNA—SMARTpool, Dharmacon) or negative control-siRNA (ON-TARGETplus Non-targeting siRNA, Dharmacon). Medium was replaced after 24 h and cells were analyzed 48 h after transfection.

### Autophagy analysis

Primary rat hepatocytes were seeded at 3.5 × 10^4^ cells per well in a 12-well plate and transfected with 50 nmol/L of Drosha-siRNA (ON-TARGETplus Rat Drosha-siRNA—SMARTpool, Dharmacon) or negative control-siRNA (ON-TARGETplus Non-targeting siRNA, Dharmacon) using Lipofectamine RNAiMax (Thermo Fisher Scientific) according to the manufacturer’s guide. After 48 h of transfection, hepatocytes were treated with chloroquine (25 µm) (Sigma) for 4 or 1 h to inhibit the autophagosome-lysosome fusion. Cells were then washed with ice-cold PBS 1× , followed by protein extraction and analysis by western blot with antibodies to LC3 (Novus Biological, dilution 1:200).

### Rapamycin treatment

Rapamycin was solubilized in DMSO, which was present in the rapamycin and control condition at a final concentration of 0.05 µl/mL. Primary rat hepatocytes were seeded at 10^6^ cells per well in a six-well plate. For short-term treatment, (100 nm) rapamycin (Calbiochem) was added to each well after 40 h from hepatocytes isolation and kept for a total of 3 h and 8 h. For long-term treatment, (10 nm), (50 nm), or (100 nm) rapamycin was added to each well just after hepatocyte isolation, for a total of 24 h or 48 h treatment. Cells were then washed with ice-cold phosphate-buffered saline or PBS 1× (Dulbecco’s PBS 10×, Gibco) before performing total protein extraction for analysis.

### Analysis of protein stability

Primary rat hepatocytes were seeded at 10^6^ cells per well in a six-well plate. 40 h after isolation, cells were treated with cycloheximide (50 µm) (Sigma) for 1, 2, and 8 h before being washed with ice-cold PBS 1 × and snap frozen for protein analysis.

### Protein lysate and quantification

Hepatocytes were washed with ice-cold PBS 1 × and scraped from dishes in 200 µL (for six-well plates) or in 100 µL (for 12-well plates) of lysis buffer (Hepes 50 mm, NaCl 150 mm, NaF 100 mm, EDTA 10 mm, Na_4_P_2_O_7_ 10 mm) supplemented with the protease inhibitors aprotinin 0.02 mg/mL (Euromedex), AEBSF 2 mm (Euromedex) and leupeptin 0.01 mg/mL (Euromedex), the phosphatase inhibitor vanadate 2 mm and 1% (v/v) Nonidet P-40. The cell suspension was then homogenized for 20 min at 4 °C and centrifuged at 14,000 g for 20 min at 4 °C. The supernatants were quantified with BCA (Interchim; Serva) and used for the experiments described below.

### Western blot analysis

Protein expression was analyzed by western blotting using the following antibodies: anti-Drosha, Cell Signaling, dilution 1:1000; anti-p70S6K, Cell Signaling, dilution 1:1000; anti-p-p70S6K (Thr389), Cell Signaling, dilution 1:1000; anti-mTOR, Cell Signaling, dilution 1:1000; anti-p-mTOR (Ser2448), Cell Signaling, dilution 1:1000; anti-β-Actin, Sigma-Aldrich, dilution 1:5000; anti-LC3, Novus Biological, dilution 1:200; MitoProfile Total OXPHOS Antibody Cocktail, MitoSciences, dilution 1:1000. An equal amount of total proteins (12–20 µg) extracted from primary rat hepatocytes were separated by electrophoresis and transferred to polyvinylidene difluoride membranes (Membrane PVDF 0.45 µm, Immobilon-P). Primary antibody incubation was performed overnight at 4 °C and then detected with HRP-conjugated anti-rabbit or anti-mouse immunoglobulins (Vector Laboratories). Immunoreactive proteins were revealed by enhanced chemiluminescence (Luminata Crescendo WB HRP Substrate, Millipore) and the intensity of each band quantified with ImageJ software (US National Institutes of Health, Bethesda, Maryland, USA).

### RNA extraction and quantitative PCR

RNA from primary hepatocytes was isolated using TRIzol reagent (Invitrogen) according to the manufacturer’s instructions. In total, 1 µg or 10 ng of total RNA was reverse transcribed into complementary DNA (cDNA) by using respectively QuantiTect Reverse Transcription Kit (Qiagen) and Universal cDNA Synthesis Kit II (Exiqon). It was then analyzed using SYBR Green (ABI PRISM 7000 Sequence Detector System). The amount of cDNA used in each reaction was normalized to the housekeeping gene cyclophilin A or miR-122-3p. The primers and real time PCR assay conditions are available upon request.

### Oxygen consumption analysis

For metabolic analysis, primary rat hepatocytes were seeded at 5 × 10^4^ cells per well in a 24 multi-well Seahorse plate (Seahorse, Agilent, Santa Clara, CA, USA). After 48 h of seeding, the extracellular acidification rate (ECAR) and the oxygen consumption rate (OCR) were determined using an XF24 Extracellular Flux Seahorse Analyzer (Agilent). Maximal OCR was determined using FCCP (2 µm), whereas rotenone and antimycin-A (2 µm each) were used to inhibit mitochondrial respiration. All parameters were calculated as described previously^40^.

### FACS analysis

Cells were detached by addition of trypsin-EDTA (0.05%), collected and either incubated for 30 min at 37 °C, 5% CO_2_ with 200 nm MitoTracker Green FM (Invitrogen), or incubated for 15 min with Annexin V and Propidium Iodide (BD Pharmingen FITC Annexin V Apoptosis Detection Kit I—BD Biosciences) at RT in the dark, according to the manufacturer’s instructions. Flow cytometric analysis was performed by FACS Calibur and analyzed by CellQuest Pro (BD).

### Immunofluorescence

Primary rat hepatocytes were seeded at 10^6^ cells per well in a six-well plate. After 48 h, cells were incubated with (50 nm) LysoTracker (Life Technologies) for 1 h at 37 °C, 5% CO_2_ and then fixed with 4% (v/v) paraformaldehyde. Incubation with anti-LC3 (Novus Biological) was performed overnight at 4 °C and detected with secondary anti-mouse (Fluo Anti-Mouse FITC, Jackson ImmunoResearch). LC3 puncta in the autophagosomes were identified by colocalization of fluorescently labeled LC3 and lysosomal markers. Images were acquired on an Axiovert microscope (Carl Zeiss, Le Pecq, France) and pictures were captured and treated with AxioVision software (Carl Zeiss).

### Mitochondrial staining

Primary rat hepatocytes were seeded at 10^6^ cells per dish in a 35 mm-diameter petri-dish coated with collagen. After 48 h, medium was replaced and cells incubated at 37 °C, 5% CO_2_ with 200 nm MitoTracker Green FM (Invitrogen). After incubation the bottom of each petri-dish was cut and reversed in a glass petri-dish. Fresh medium was added and mitochondria visualized by excitation with 516 nm wavelength. Images were acquired with an observer D1 microscope (Carl Zeiss GmbH, Jena, Germany) under oil immersion and pictures were captured and treated with Zen software (Carl Zeiss).

### Terminal deoxynucleotidyl transferase mediated dUPT nick-end labeling (TUNEL) assay

The apoptotic rate of primary rat hepatocytes was detected by using the In Situ Cell Death Detection Kit, Fluorescein (Roche). Cells were fixed by incubation with ice-cold MetOH 100% for 1 h at 4 °C. Afterwards, they were washed twice with PBS and incubated with TUNEL reaction mixture for 1 h at 37 °C, as described by the manufacturer. Positive cells were visualized by excitation with 515-565 nm wavelength and quantified with ImageJ software. Images were acquired on an Observer D1 microscope (Carl Zeiss GmbH, Jena, Germany) using an objective A-Plan × 10/0.25 Ph 1.

### Statistical analysis

Data are presented as mean ± SEM; *n* represents the number of independent rats. Statistical analysis was performed using GraphPad Prism version 6.01 for Windows (GraphPad Software, La Jolla California USA). Paired two-tail Student’s *t* test was used to compare two conditions. Differences between more than two conditions were analyzed by one-way analysis of variance (paired or unpaired) followed by Sidak’s multiple comparison test. A *p* value < 0.05 was considered significant.

## Supplementary information

Supplemental figures

Supplemental figure legends

Uncropped Western Blots Figure 6-7-8

Uncropped Western Blots Figure 5

Uncropped Western Blots Figure 1-3-4

## References

[1] Wang ZM (2003). Total body protein: a new cellular level mass and distribution prediction model. Am. J. Clin. Nutr..

[2] Elwyn DH, Parikh HC, Shoemaker WC (1968). Amino acid movements between gut, liver, and periphery in unanesthetized dogs. Am. J. Physiol..

[3] Wahren J, Felig P, Hagenfeldt L (1976). Effect of protein ingestion on splanchnic and leg metabolism in normal man and in patients with diabetes mellitus. J. Clin. Invest..

[4] Lardy HA, Shrago E, Young JW, Paetkau V (1964). The pathway of gluconeogenesis in liver. Science.

[5] Schimassek H, Gerok W (1965). Control of the levels of free amino acids in plasma by the liver. Biochem. Z..

[6] Michalopoulos GK, DeFrances M (1997). Liver regeneration. Science.

[7] Gehart H, Clevers H (2015). Repairing organs: lessons from intestine and liver. Trends Genet..

[8] Kmieć Z (2001). Cooperation of liver cells in health and disease. Adv. Anat. Embryol. Cell Biol..

[9] Mangnall D, Bird NC, Majeed AW (2003). The molecular physiology of liver regeneration following partial hepatectomy. Liver Int..

[10] Malato Y (2011). Fate tracing of mature hepatocytes in mouse liver homeostasis and regeneration. J. Clin. Invest..

[11] Kim E, Goraksha-Hicks P, Li L, Neufeld TP, Guan KL (2008). Regulation of TORC1 by Rag GTPases in nutrient response. Nat. Cell Biol..

[12] Sancak Y (2008). The rag GTPases bind raptor and mediate amino acid signaling to mTORC1. Science.

[13] Hara K (1998). Amino acid sufficiency and mTOR regulate p70 S6 kinase and eIF-4E BP1 through a common effector mechanism. J. Biol. Chem..

[14] Christie GR, Hajduch E, Hundal HS, Proud CG, Taylor PM (2002). Intracellular sensing of amino acids in Xenopus laevis oocytes stimulates p70 S6 kinase in a target of rapamycin-dependent manner. J. Biol. Chem..

[15] Russell RC, Fang C, Guan K-L (2011). An emerging role for TOR signaling in mammalian tissue and stem cell physiology. Development.

[16] Ye P (2015). An mTORC1-Mdm2-Drosha axis for miRNA biogenesis in response to glucose- and amino acid-deprivation. Mol. Cell.

[17] Meister G (2004). Human Argonaute2 mediates RNA cleavage targeted by miRNAs and siRNAs. Mol. Cell.

[18] Holec M (1999). Nutritional modulation of liver regeneration by carbohydrates, lipids, and amino acids: a review. Nutrition.

[19] Gomes SP (2017). Stereology shows that damaged liver recovers after protein refeeding. Nutrition.

[20] Werner-Washburne M, Braun E, Johnston GC, Singer RA (1993). Stationary phase in the yeast Saccharomyces cerevisiae. Microbiol. Rev..

[21] Gray JV (2004). “Sleeping Beauty”: quiescence in Saccharomyces cerevisiae. Microbiol. Mol. Biol. Rev..

[22] Grisham JW (1962). A morphologic study of deoxyribonucleic acid synthesis and cell proliferation in regenerating rat liver; autoradiography with thymidine-H8. Cancer Res..

[23] Cheshier SH, Morrison SJ, Liao X, Weissman IL (1999). In vivo proliferation and cell cycle kinetics of long-term self-renewing hematopoietic stem cells. Proc. Natl Acad. Sci. USA.

[24] Orford KW, Scadden DT (2008). Deconstructing stem cell self-renewal: genetic insights into cell-cycle regulation. Nat. Rev. Genet..

[25] Kim M, Cooper DD, Hayes SF, Spangrude GJ (1998). Rhodamine-123 staining in hematopoietic stem cells of young mice indicates mitochondrial activation rather than dye efflux. Blood.

[26] Siggins RW, Zhang P, Welsh D, LeCapitaine NJ, Nelson S (2008). Stem cells, phenotypic inversion, and differentiation. Int J. Clin. Exp. Med..

[27] Yhee JY (2017). The effects of collagen-rich extracellular matrix on the intracellular delivery of glycol chitosan nanoparticles in human lung fibroblasts. Int. J. Nanomed..

[28] Martinez I (2017). An Exportin-1–dependent microRNA biogenesis pathway during human cell quiescence. PNAS.

[29] Tang X (2013). Acetylation of drosha on the N-terminus inhibits its degradation by ubiquitination. PLoS ONE.

[30] Kendrick AA (2011). Fatty liver is associated with reduced SIRT3 activity and mitochondrial protein hyperacetylation. Biochem. J..

[31] Meyer JG (2018). Temporal dynamics of liver mitochondrial protein acetylation and succinylation and metabolites due to high fat diet and/or excess glucose or fructose. PLoS ONE.

[32] Dalmay T (2006). Short RNAs in environmental adaptation. Proc. R. Soc. B Biol. Sci..

[33] Wang J, Cui Q (2012). Specific roles of MicroRNAs in their interactions with environmental factors. J. Nucleic Acids.

[34] Nawrot TS, Vrijens K, Bollati V (2015). MicroRNAs as potential signatures of environmental exposure or effect: a systematic review. Environ. Health Perspect..

[35] Voskarides K (2017). Plasticity vs mutation. the role of microRNAs in human adaptation. Mech. Ageing Dev..

[36] Höck J, Meister G (2008). The Argonaute protein family. Genome Biol..

[37] Benjamin C, Gregory J (2011). Hannon. Small RNA sorting: matchmaking for Argonautes. Nat. Rev. Genet.

[38] Liu J (2004). Argonaute2 is the catalytic engine of mammalian RNAi. Science.

[39] Fehlmann M, Le Cam A, Freychet P (1979). Insulin and glucagon stimulation of amino acid transport in isolated rat hepatocytes. Synthesis of a high affinity component of transport. J. Biol. Chem..

[40] Brand MD, Nicholls DG (2011). Assessing mitochondrial dysfunction in cells. Biochem. J..

