# Supplementary material for: Amino acid-induced regulation of hepatocyte growth: possible role of Drosha
Source: Cell Death Dis. 2019 Jul 22;10(8):566. doi: 10.1038/s41419-019-1779-7 (PMC6646398; doi:10.1038/s41419-019-1779-7)
Supplement: Supplementary file 1 — Supplemental figures [file 41419_2019_1779_MOESM1_ESM.pptx]

## Slide 1
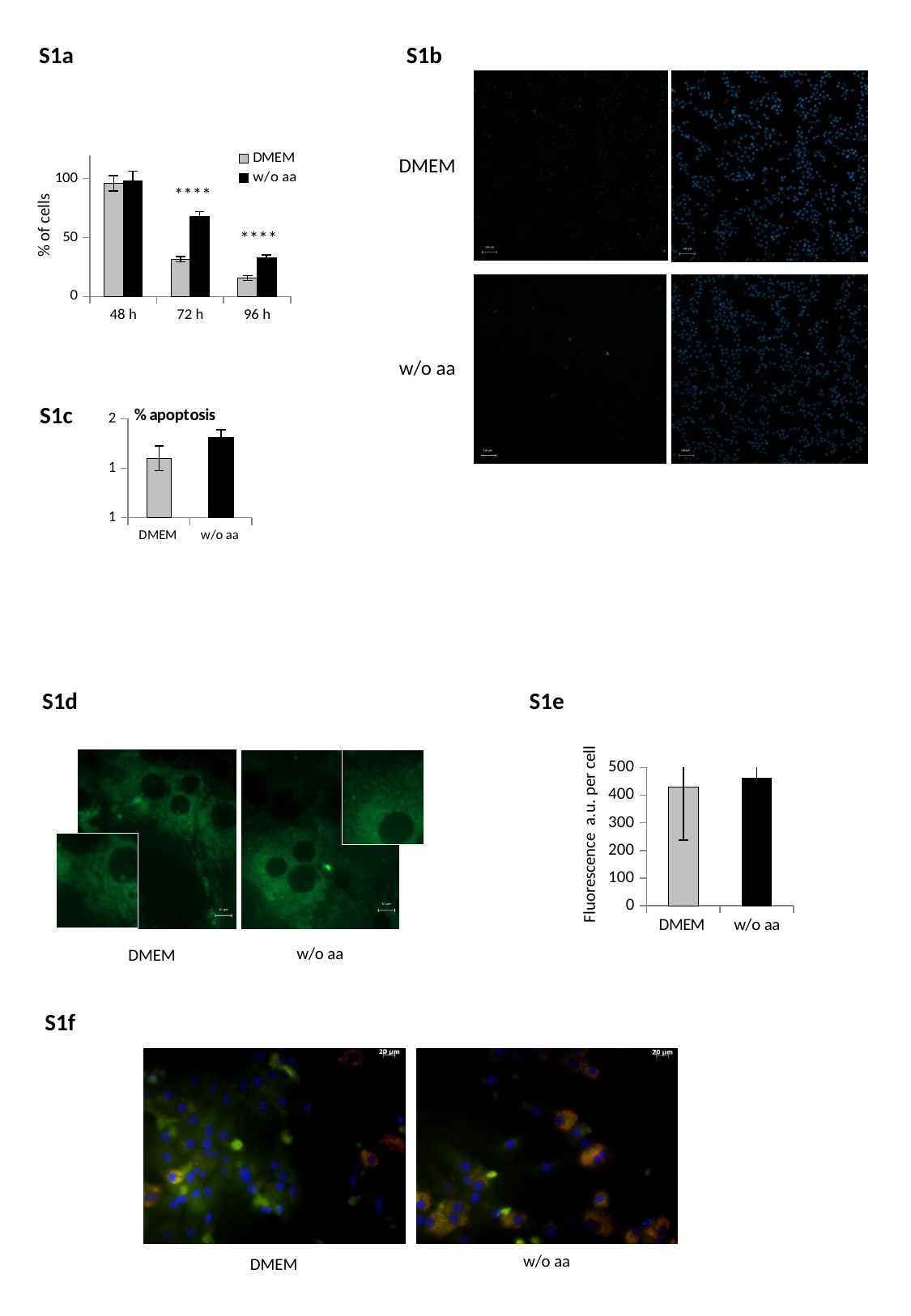

S1a
S1b
### Chart
| Category | DMEM | w/o aa |
|---|---|---|
| 48 h | 96.11695906432747 | 98.15972222222223 |
| 72 h | 31.59766081871345 | 68.10416666666667 |
| 96 h | 15.615037593984963 | 32.776785714285715 |DMEM
****
% of cells
****
w/o aa
S1c
### Chart:
| Category | % apoptosis |
|---|---|
| DMEM | 1.0997210939817965 |
| w/o aa | 1.3096170678708394 |S1d
S1e
### Chart
| Category | |
|---|---|
| DMEM | 430.2 |
| w/o aa | 462.154 |Fluorescence a.u. per cell
w/o aa
DMEM
S1f
w/o aa
DMEM

## Slide 2
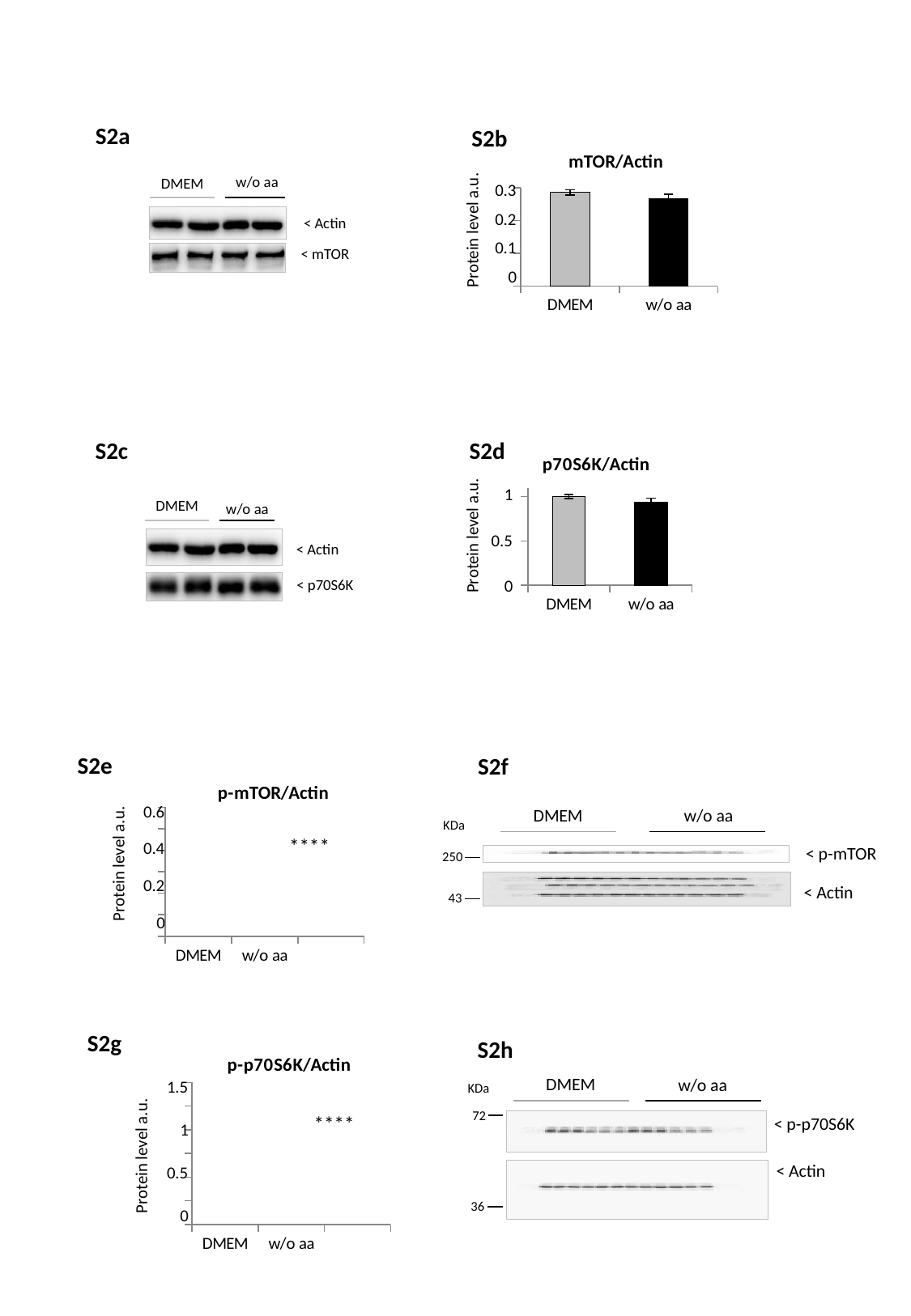

S2a
S2b
### Chart:
| Category | mTOR/Actin |
|---|---|
| DMEM | 0.28610826731932476 |
| w/o aa | 0.26676648663533875 |w/o aa
DMEM
0.3
0.2
0.1
0
< Actin
Protein level a.u.
< mTOR
S2c
S2d
### Chart:
| Category | p70S6K/Actin |
|---|---|
| DMEM | 1.0023328519519512 |
| w/o aa | 0.9411881061735309 |1
0.5
0
DMEM
w/o aa
Protein level a.u.
< Actin
< p70S6K
S2e
S2f
### Chart:
| Category | p-mTOR/Actin |
|---|---|
| DMEM | 0.38218410280807347 |
| w/o aa | 0.2569085305805789 |0.6
0.4
0.2
0
DMEM
w/o aa
KDa
****
< p-mTOR
250
Protein level a.u.
< Actin
43
S2g
### Chart:
| Category | p-p70S6K/Actin |
|---|---|
| DMEM | 1.1875656814843603 |
| w/o aa | 0.5703731373592712 |S2h
DMEM
w/o aa
1.5
1
0.5
0
KDa
72
****
< p-p70S6K
Protein level a.u.
< Actin
36

## Slide 3
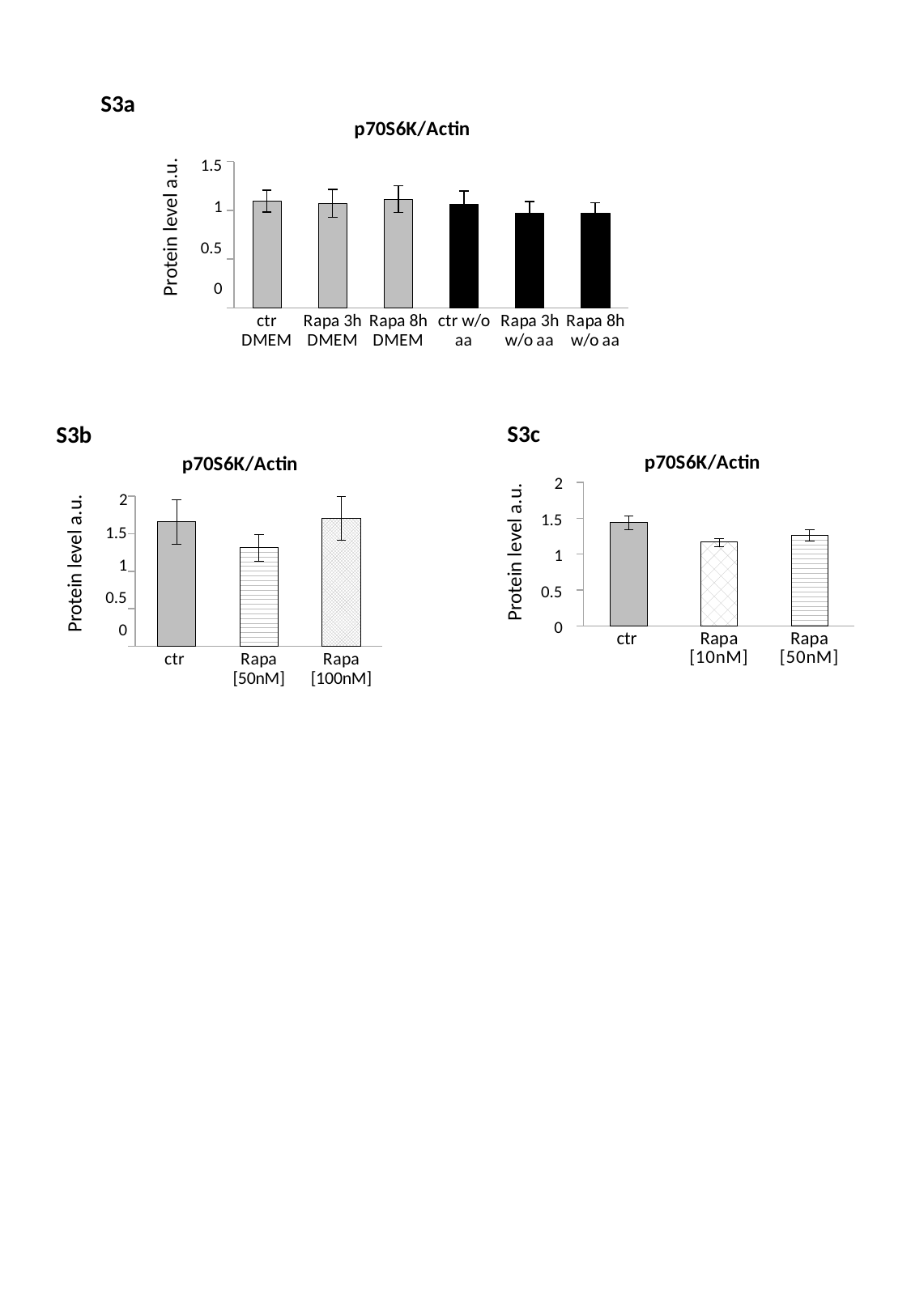

S3a
### Chart:
| Category | p70S6K/Actin |
|---|---|
| ctr DMEM | 1.0949947965675768 |
| Rapa 3h DMEM | 1.0712420272847716 |
| Rapa 8h DMEM | 1.1155787120948661 |
| ctr w/o aa | 1.059632187711611 |
| Rapa 3h w/o aa | 0.9685647338347566 |
| Rapa 8h w/o aa | 0.9684323775404335 |1.5
1
0.5
0
Protein level a.u.
S3c
S3b
### Chart:
| Category | p70S6K/Actin |
|---|---|
| ctr | 1.6585240052836958 |
| Rapa [50nM] | 1.3173498648916373 |
| Rapa [100nM] | 1.7073223246821734 |
### Chart:
| Category | p70S6K/Actin |
|---|---|
| ctr | 1.4381053346861836 |
| Rapa [10nM] | 1.166697393444741 |
| Rapa [50nM] | 1.2638876604185298 |2
1.5
1
0.5
0
2
1.5
1
0.5
0
Protein level a.u.
Protein level a.u.

## Slide 4
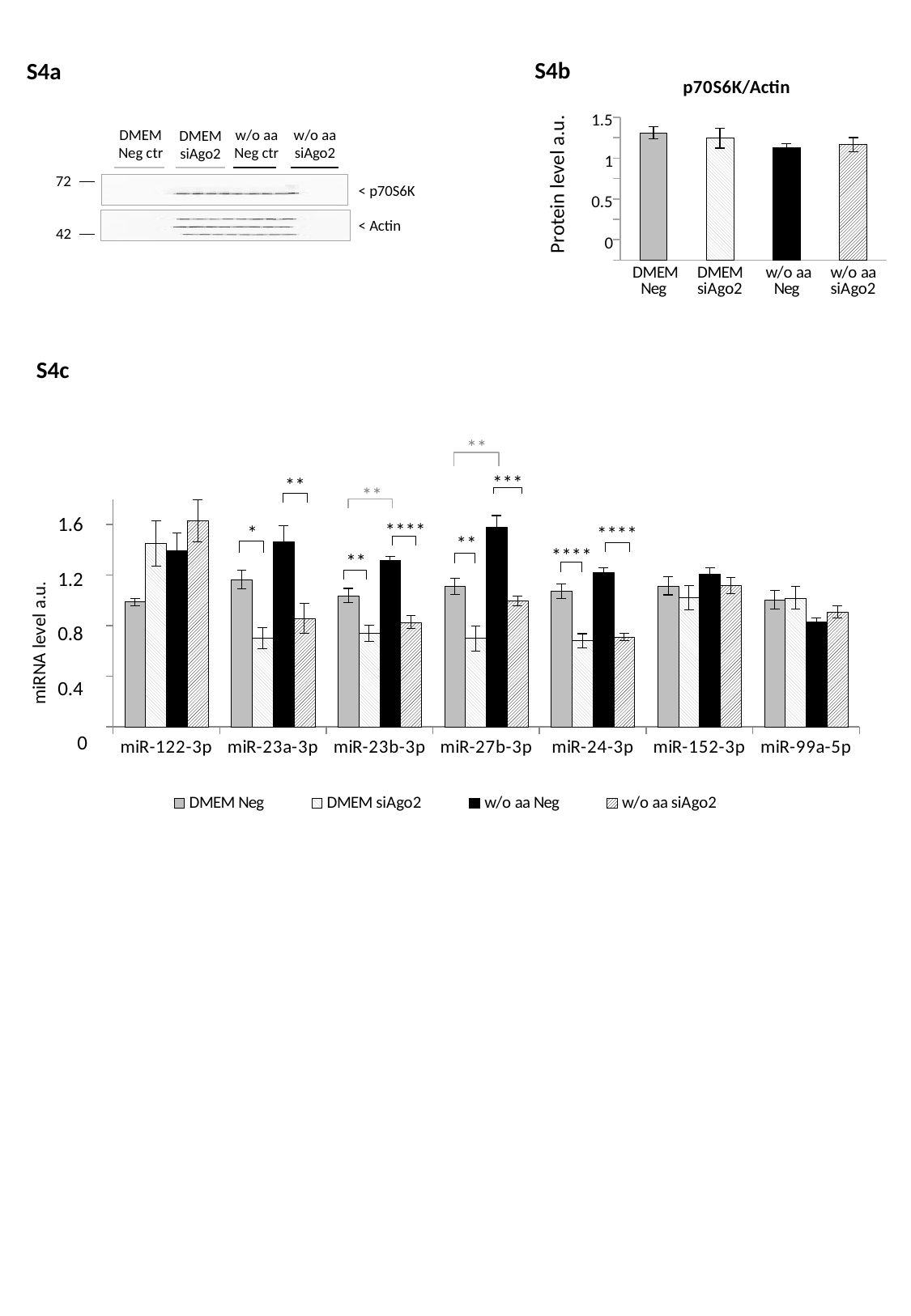

S4b
S4a
### Chart:
| Category | p70S6K/Actin |
|---|---|
| DMEM Neg | 1.248266463214563 |
| DMEM siAgo2 | 1.1963882762115599 |
| w/o aa Neg | 1.0993512575352116 |
| w/o aa siAgo2 | 1.1316415191460543 |1.5
1
0.5
0
DMEM
Neg ctr
w/o aa
Neg ctr
w/o aa
siAgo2
DMEM
siAgo2
72
Protein level a.u.
< p70S6K
< Actin
42
S4c
**
***
**
**
### Chart
| Category | DMEM Neg | DMEM siAgo2 | w/o aa Neg | w/o aa siAgo2 |
|---|---|---|---|---|
| miR-122-3p | 0.9891274221487688 | 1.451393213045482 | 1.394783544129327 | 1.6303661425519753 |
| miR-23a-3p | 1.1652758978657813 | 0.701505581368535 | 1.4649399665008929 | 0.8570424215874395 |
| miR-23b-3p | 1.0393202320929629 | 0.7397641048427613 | 1.3163744181231112 | 0.8275230839718509 |
| miR-27b-3p | 1.110266256465359 | 0.7009104661046229 | 1.5782891229700622 | 0.99490225613943 |
| miR-24-3p | 1.0731086762809408 | 0.6814856769415765 | 1.2215711998687369 | 0.7099976264909573 |
| miR-152-3p | 1.1151058606560278 | 1.0213442665864383 | 1.2100405526324065 | 1.1169959382186416 |
| miR-99a-5p | 1.00451223500206 | 1.0202459743454186 | 0.8317052089669076 | 0.909941446156661 |
1.6
1.2
0.8
0.4
0
****
*
****
**
****
**
miRNA level a.u.
