# Supplementary material for: Amino acid-induced regulation of hepatocyte growth: possible role of Drosha
Source: Cell Death Dis. 2019 Jul 22;10(8):566. doi: 10.1038/s41419-019-1779-7 (PMC6646398; doi:10.1038/s41419-019-1779-7)
Supplement: Supplementary file 2 — Supplemental figure legends [file 41419_2019_1779_MOESM2_ESM.docx]

**Figure S1. AaD in primary adult rat hepatocytes reduces the drop in cell number.** (a) Cell counting of primary adult rat hepatocytes in full-aa medium (DMEM) and in absence of aa (w/o aa) at different time points. Results are normalized to values at 48 h for both experimental conditions and expressed in percentage, being 48 h-cell number 100%. Values are means ± SEM (n=3). ****p < 0.0001. (b) Detection of apoptosis by Tunel assay: apoptotic cells (green) were counted together with DAPI (blue) in fixed primary adult rat hepatocytes after 48 h in DMEM or in medium w/o aa. Scale bar = 100 µm. (c) Quantification of primary adult rat hepatocyte’s apoptotic rate in DMEM and after 48 h aaD (w/o aa) by Tunel assay. Results are expressed in percentage of apoptotic cells over total cell number. Values are means ± SEM (n=3). (d) Mitochondrial network organization observed in control (DMEM) and aa-deprived (w/o aa) hepatocytes after 48 h from plating using MitoTracker Green FM labeling. Scale bar = 10 µm. (e) FACS analysis of the mean intensity of fluorescence per cell in primary adult rat hepatocytes after 48 h in DMEM or w/o aa using MitoTracker Green FM labeling. Values are means ± SEM (n=3). (f) Detection of LC3 puncta in the autophagosomes of primary adult rat hepatocytes after 48h aaD: cells in autophagy are characterized by co-localization of LC3 puncta (green) and lysosomal marker (red). Cell nuclei are marked with DAPI (blue). Scale bar = 20µm.

**Figure S2. 48 h aaD in primary adult rat hepatocytes reduces mTOR and p70S6K phosphorylation but does not affect the mTOR and the p70S6K total protein level.** (a) Western blot analysis and (b) quantification of mTOR protein level after 48 h in a full-aa medium (DMEM) or in medium w/o aa. Values are means ± SEM (n=3). (c) Western blot analysis and (d) quantification of p70S6K protein level after 48h in DMEM or in medium w/o aa. Values are means ± SEM (n=3). (e-f) Western blot analysis (e) and quantification (f) of mTOR phosphorylation after 48h in a full-aa medium (DMEM) or in medium w/o aa. Values are means ± SEM (n=7). ****p < 0.0001 (g-h) Western blot analysis (g) and quantification (h) of p70S6K phosphorylation after 48 h in a DMEM or w/o aa. Values are means ± SEM (n=15). ****p < 0.0001

**Figure S3. Neither short nor long rapamycin treatment affect total p70S6K protein level.** (a) Western blot quantification of p70S6K protein in primary adult rat hepatocytes after 48 h aaD without any treatment (Rapa ctr), after 3 h and 8 h rapamycin treatment [100 nM] (Rapa 3h, Rapa 8h). Rapamycin was solubilized in DMSO, which was present in the rapamycin and control condition at a final concentration of 0.05 µl/mL. Values are means ± SEM (n=3). (b) Western blot quantification of p70S6K protein in aa-deprived hepatocytes without any treatment (ctr) and rapamycin treatment for 24 h. Values are means ± SEM (n=3). (c) Western blot quantification of p70s6K protein in aa-deprived hepatocytes without any treatment (ctr) and rapamycin treatment for 48 h. Values are means ± SEM (n=3).

**Figure S4. Ago2-knockdown does not affect total p70S6K protein level.** (a-b) Western blot analysis (a) and quantification (b) of p70S6K total protein in primary adult rat hepatocytes transfected with 50 nmol/L negative control-siRNA (Neg. Ctr) or Ago2-siRNA (siAgo2) for 48 h in full-aa medium (DMEM) or in absence of aa (w/o aa). Values are means ± SEM (n=3). (c) Analysis of miRNA expression level in primary adult rat hepatocytes transfected with 50 nmol/L Neg. Ctr or siAgo2 for 48 h in DMEM or medium w/o aa. RNA was extracted, reverse transcribed and analyzed by Real Time PCR. The expression of miR-23a-3p, -23b-3p, -27b-3p, -24-3p, -152-3p, -99a-5p was normalized to the miR-122-3p transcript level, while the expression of miR-122-3p was normalized to miR-99a-5p. Values are means ± SEM (n=3). *p < 0.05, **p < 0.005, ***p < 0.0005, ****p < 0.0001
